# A parametric geometry model of the aortic valve for subject-specific blood flow simulations using a resistive approach

**DOI:** 10.1007/s10237-023-01695-5

**Published:** 2023-02-28

**Authors:** Giorgia Pase, Emiel Brinkhuis, Tanja De Vries, Jiří Kosinka, Tineke Willems, Cristóbal Bertoglio

**Affiliations:** 1 Bernoulli Institute, University of Groningen, Nijenborgh 9, Groningen, 9747AG The Netherlands; 2 Department of Radiology, University Medical Center Groningen, University of Groningen, Hanzeplein 1, Groningen, 9713GZ The Netherlands

**Keywords:** Parametric model, Aortic valve model, Subject-specific, Blood flow simulation

## Abstract

Cardiac valves simulation is one of the most complex tasks in cardiovascular modeling. Fluid–structure interaction is not only highly computationally demanding but also requires knowledge of the mechanical properties of the tissue. Therefore, an alternative is to include valves as resistive flow obstacles, prescribing the geometry (and its possible changes) in a simple way, but, at the same time, with a geometry complex enough to reproduce both healthy and pathological configurations. In this work, we present a generalized parametric model of the aortic valve to obtain patient-specific geometries that can be included into blood flow simulations using a resistive immersed implicit surface (RIIS) approach. Numerical tests are presented for geometry generation and flow simulations in aortic stenosis patients whose parameters are extracted from ECG-gated CT images.

## Introduction

Aortic stenosis is the most common valvular disease (Coffey et al. 2016). It consists in a thickening (inflammation) and a progressive stiffening (calcification) of the aortic valve leaflets, limiting their mobility, narrowing the valve opening and hence obstructing the blood flow from the heart to the systemic circulation.

Constitutive relations of the valve tissue are complex and variable per subject, leaflet and valve, in particular related to its complex fiber structure (Heyden et al. 2015). Moreover, experimental testing of valves does not fully reproduce the loading conditions in vivo : while ex-vivo setups apply a pure traction loading, the valve in-vivo actually bends hence entailing both traction and compression. Therefore, *truly subject-specific* fluid-solid interaction (FSI) simulations are not feasible in spite of important advances in the numerical solution of the FSI problem (Astorino et al. 2009; Annese et al. 2022; Kaiser et al. 2021; Burman et al. 2022; Fernández and Gerosa 2021). From the point of view of applications, FSI computations have primarily focused on the influence of valve shape and properties in the blood flow dynamics (Marom et al. 2013; Mohammadi et al. 2017; Xu et al. 2018; Kaiser et al. 2022; Viola et al. 2021; Lee et al. 2021).

Also with the focus on studying the effect of the valve shape on hemodynamic quantities, rigid-walls (and mostly steady state) simulations of valvular flows at open positions have been performed instead of FSI in order to reduce computational complexity (Hellmeier et al. 2018; Weese et al. 2017; Franke et al. 2020; Hoeijmakers et al. 2019, 2020, 2021). However, this requires advanced meshing procedures since valve geometries must be defined a priori within the boundary of the mesh.

When valves need to be incorporated within large cardiac (fluid-)mechanics simulations, it was originally proposed in Astorino et al. (2012) to include them as *resistive immersed surfaces* (RIS). With this approach, the valve shape needs to be still accounted in the computational mesh but now as an internal surface within the flow domain. Then, on the immersed surface, the velocity is made very small on the valve by penalization, and the pressure is let to jump by doubling the degrees of freedom for the pressure on the surface. The main advantage over modeling the valve in the mesh itself is that with RIS several valve configurations can be included, and they can be turned on and off *during* the computations, for instance depending on the pressure and flow conditions. This approach was recently extended to include additional information on the pressure when several valves are modeled and isovolumetric phases are computed (This et al. 2020).

In order to completely remove the influence of the valve shape on the fluid mesh, several authors proposed to include valves as *resistive immersed implicit surfaces* (RIIS), i.e., in terms of a resistive volumetric function (Laadhari and Quarteroni 2016; Fedele et al. 2017; Fumagalli et al. 2020; Fuchsberger et al. 2022; Fumagalli 2021), which converges to the problem with a fixed obstacle when the value of the resistance increases as proven in (Aguayo and Lincopi 2022). The position of the valves can be therefore determined without defining them in the mesh *a priori*. To do so, a distance function needs to be constructed, which is then thresholded to represent the valve with a certain thickness.

In Fuchsberger et al. (2022), the RIIS valve geometry was modeled in closed positions as a slab separating all cardiac chambers (and just removed when the valves are supposed to open). In Laadhari and Quarteroni (2016); Fedele et al. (2017); Fumagalli et al. (2020); Fumagalli (2021), the RIIS valve geometry was modeled using a polynomial surface of a certain degree to represent the distance function, which then needs to be cut using additional surfaces. In Fedele et al. (2017), the polynomial coefficients of the valve surface were fitted for a single patient by selecting a large amount of control points in a 3D CT image. To the best of our knowledge, the application of RIIS to patient-specific, and in particular pathological, shapes has not been reported yet.

An alternative way to construct the 3D valve geometry model is to use a low-dimensional parametric representation of the shape (i.e., by quantities like radii, angles, etc.). The low parametric dimension in these models allows to extract the 3D geometry with a reduced number of control points and/or measurements, therefore considerably easing the segmentation process. Moreover, it could have the potential to segment the 3D shape from a set of 2D images.

Parametric models have been originally devoted to obtaining shape designs for prosthetic valves, hence aiming to reproduce healthy valve geometries, see e.g., Swanson and Clark (1974); Thubrikar et al. (1981); Labrosse et al. (2006). Recently in Haj-Ali et al. (2012), a more general model for the boundary curves defining each half-leaflet was proposed and assessed in one 3D-TEE measurement of a non-pathological valve by assuming inter- and intra-leaflet symmetry. Therefore, to the best of the authors’ knowledge, parametric models in the context of subject-specific, possibly pathological valve shapes has remained unexplored. In particular, in the context of blood flow simulations, parametric models require to be formulated in terms of parametric surfaces in order to compute distance functions to generate the RIIS models.

In this work, we propose a generalization of the model from Haj-Ali et al. (2012) such that surfaces can be constructed in a parametric way and distance functions to the valves can be computed for RIIS-based modeling. We also propose relaxing the inter-symmetry in the leaflets in order to adapt the shape of the valve to a wider range of subjects. Doing so we aim to be capable of generating pathological geometries in a wide range of shapes. We propose an approach to generate resistive volume models from the valvular geometry to incorporate them into fluid flow simulations. We assessed the capability of the model to generate a wide variety of shapes on 10 open valve geometries coming from CT images before a transcatheter aortic valve intervention (TAVI). Examples of transvalvular blood flow simulations using resistive volumes are also included. The shape parameters extraction protocol from 3D CT images is also explained.

## The 3D parametric aortic valve model

A normal aortic valve consists of three leaflets which can open and close to control the blood flow through it. Our 3D parametric model aims to reproduce the valve shape to obtain a geometry that is as realistic as possible. With this goal in mind, as mentioned above we based our work on the valve model described in Haj-Ali et al. (2012) and we added some important improvements to have more control on the way the leaflets bend when the valve opens. For the sake of completeness, we present here the full model in detail, mentioning explicitly when changes are performed.

The native tricuspid aortic valve has a complex 3D structure that can be represented defining parametric curves (enclosing different surfaces) that allow us to shape up the leaflets and to obtain a realistic description of these structures. The reference model we improved describes the valve assuming three identical cusps (inter-symmetry assumption) with internal symmetry (intra-symmetry assumption, i.e., each leaflet is composed by two identical halves), limiting the construction effort to half of a single cusp which is then replicated identically and rotated to obtain the tricuspid structure. With such assumptions it is indeed possible to obtain a good general approximation of the aortic valve, but they end up to be too strict to properly represent a subject-specific valve, especially when referring to pathological cases. So, in order to have a geometric representation that better adapts to human anatomy, the inter-symmetry assumption has been relaxed. Thanks to this choice, we are able to describe valves with three completely different leaflets that better fit the shape we see from ECG-gated CT images.

**Fig. 1:**
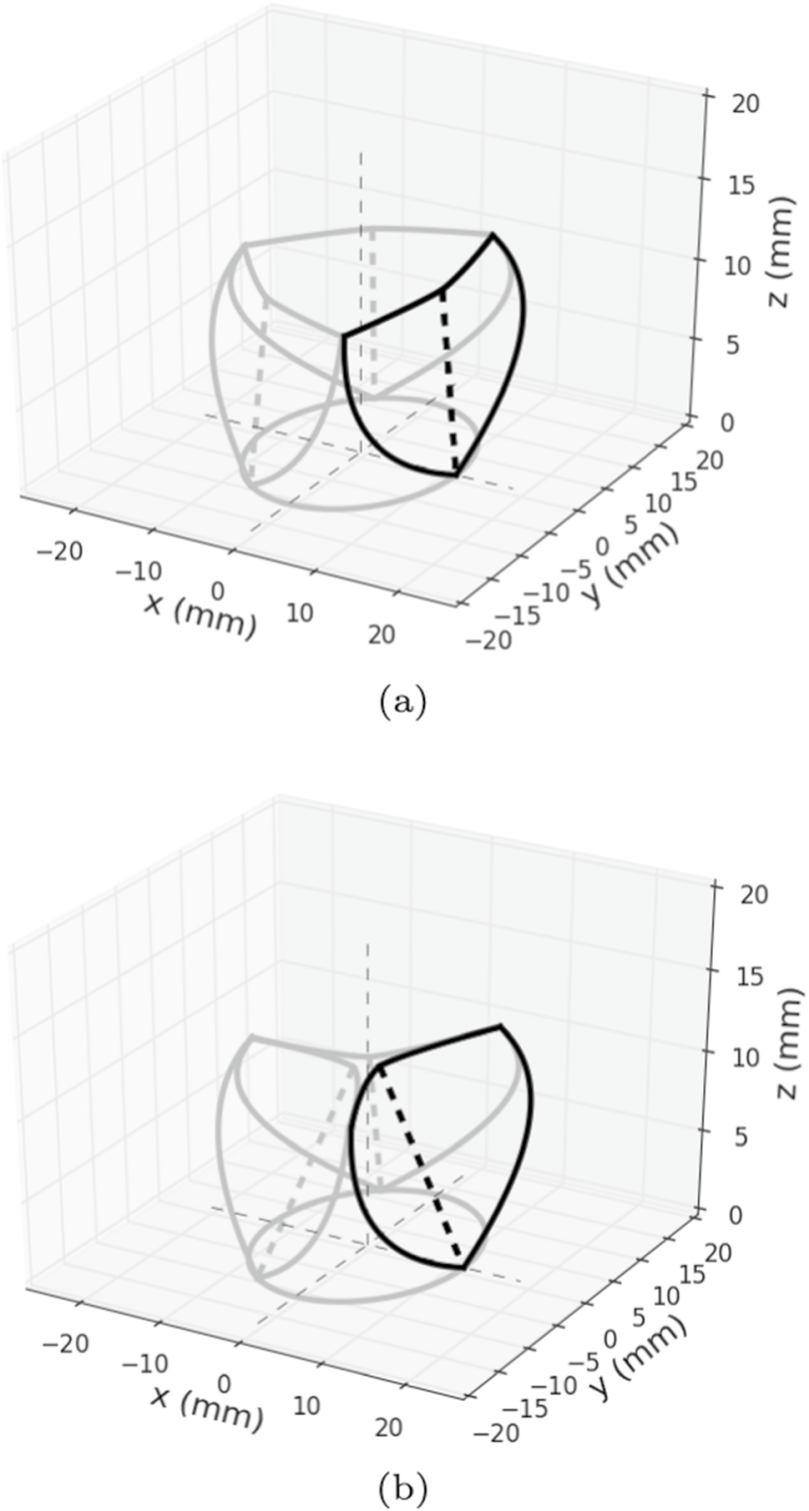
Basic 3D example of the aortic valve geometry we want to obtain for our simulations in open (1a) and closed (1b) position. Here, inter- and intra-leaflet symmetry are enforced

Moreover, since at this stage of our work we are not interested in recovering the sinuses and the interleaflet triangles (see Fig. 1), we need to replace these structures with a completely new curve to enclose the analytical surfaces that otherwise would remain open. From now on, we refer to such curve as the *sinus curve*. Another structural improvement consists in the introduction of a new curve, that we called the *bending curve*, to better describe the way the leaflet bends when opening. Such improvements of the reference model forced us to modify the way we recover the 3D final surface, dividing the leaflet into two parts connecting at the bending curve. This curve, as long as the details on the generation of the 3D surface, is introduced and explained in full detail in the following section.

### Curves definition

**Fig. 2:**
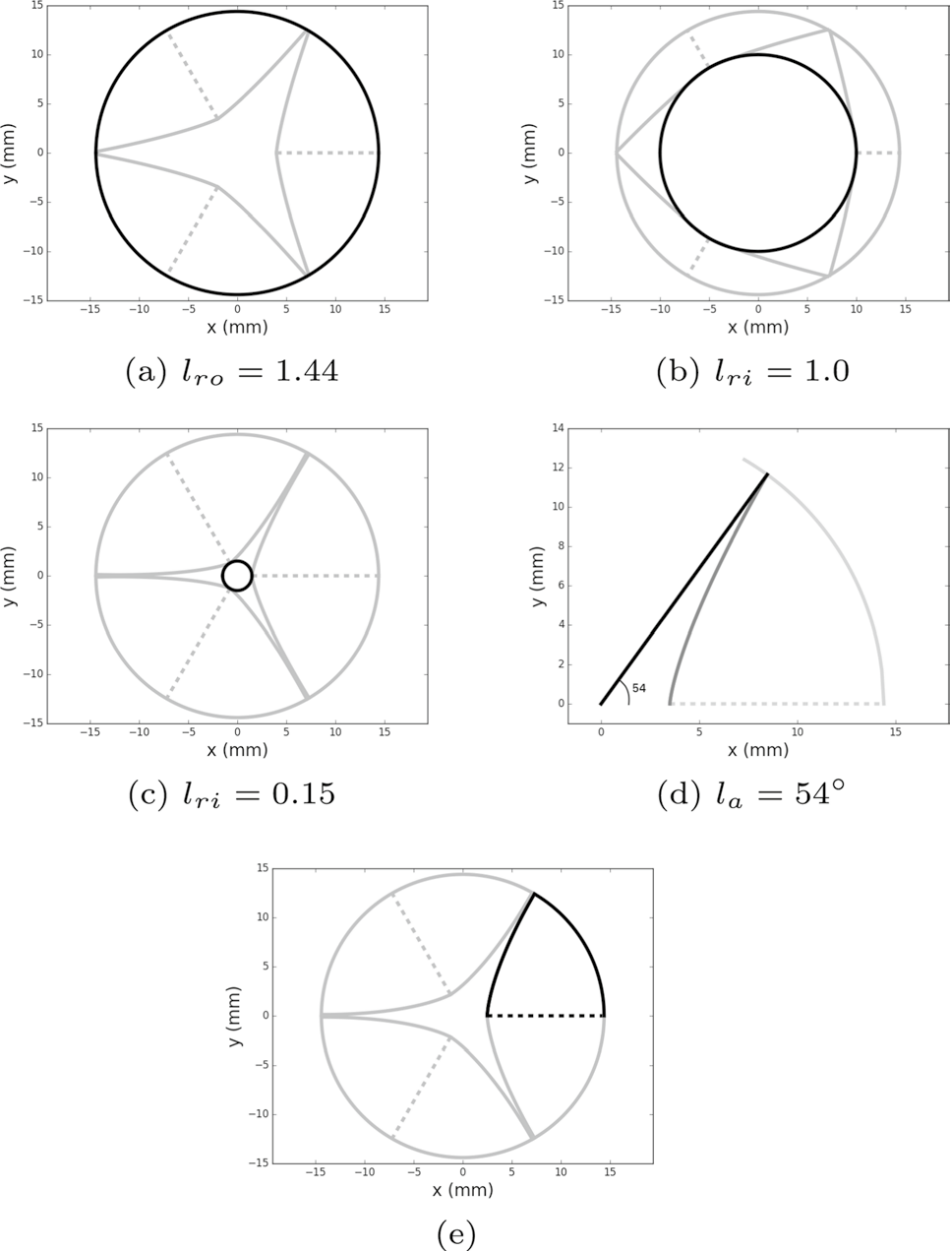
The outer circle’s radius, $l_{ro}$, represents the size of the aorta and the inner circle’s radius, $l_{ri}$, determines the valve’s state (the bigger the measurement, the more open the valve). The leaflet angle $l_a$ determines the half leaflet region of interest highlighted in the last picture

We here define the 4 curves we need to create the 3D surface of the leaflet, namely the *leaflet curve*, the *bending curve*, the *symmetry curve*, and the *sinus curve*. We assume the valve is included in a cylinder within a (*x*, *y*, *z*) coordinate system, with *z* the axial coordinate. As mentioned above, we relaxed the inter-symmetry hypothesis but we kept considering each leaflet as a symmetric surface with respect to its own axis. So, likewise Haj-Ali et al. (2012), the parametric description is limited to one sixth of the entire geometry, thus in the $0 \le \theta \le l_a$ region, where $l_a$ is what we refer to as the *leaflet angle* (see Fig. 2d). Since we relaxed the inter-symmetry assumption, the leaflet angle value is not fixed, allowing us to generate a final model with three different leaflets. The region of interest is shown in Fig. 2e.

#### Leaflet curve

Consider the 2D coordinate system (*x*, *y*) crossing the axis of the valve structure at $z=l_h$, where $l_h$ stands for the *leaflet height*. Looking at the valve from this perspective, it is possible to identify the physical parameters we need to give the analytical description of the *leaflet curve*, i.e., the free edge of the leaflet: the radius of the aorta at $z=l_h$ ($l_{ro}$, Fig. 2a), the radius of the inner circle touching the leaflet at the symmetry line, and describing the state of the valve ($l_{ri}$, Fig. 2b, c) and the leaflet angle ($l_a$, Fig. 2d).

We can now define the leaflet curve as in Haj-Ali et al. (2012). Let the leaflet curve be defined as a $x=f(y)$ type equation. The curve is connected to the inner circle at $(l_{ri}, 0)$, at the symmetry line. Since the curve will be reflected to complete the leaflet, we impose the derivative at $y=0$ to be equal to zero, in order to have a smooth transition. Finally, the curve intersects the outer circle in a point that can be represented using the parameters defined above. All these statements result in the following conditions:

$$\begin{aligned} f(0)&= l_{ri}\,\text {,}\\ f'\,(0)&= 0\,\text {,}\\ f(l_{ro} \cdot \sin l_{a})&= l_{ro} \cdot \cos l_{a}\,\text {.} \end{aligned}$$

Solving for these three conditions, we obtain the following formula for *f*, as given in Haj-Ali et al. (2012):

1 $$\begin{aligned} f(y) = l_{ri} + \alpha y^{l_p}\,\text {,} \end{aligned}$$

with

$$\begin{aligned} \alpha = \frac{l_{ro} \cdot \cos l_{a}-l_{ri}}{(l_{ro} \cdot \sin l_a)^{l_p}}\,\text {.} \end{aligned}$$

In this expression, we use a new parameter, the *leaflet power* $(l_p)$, which describes the form of the leaflet curve.

Finally, if we consider another 2D local coordinate system (*x*, *y*) at $z=0$, we are able to identify the lowest point where the cusps are connected to the valve structure, which we refer to as the *aortic annulus*, and to extract its radius, $r_r$.

#### Bending curve

After describing the valve geometry at the top and bottom level, we are now going to define other three curves to enclose the 3D surface of the leaflet. As mentioned above, we added the *bending curve* to the model in Haj-Ali et al. (2012). The definition is equal to the leaflet curve’s one but the parameters involved are defined at $z = b_h$, where $b_h$ stands for the *bending height*, which is generally located halfway between the annulus and the top of the valve. Let the bending curve be defined as a $x=f(y)$ type equation, then

2 $$\begin{aligned} g(y) = b_{ri} + \beta y^{b_p}\,\text {,} \end{aligned}$$

where

$$\begin{aligned} \beta = \frac{b_{ro} \cdot \cos b_{a}-b_{ri}}{(b_{ro} \cdot \sin b_a)^{b_p}}. \end{aligned}$$

The parameters involved are analogous to the leaflet ones: the radius of the aorta at $z=b_h$ ($b_{ro}$), the radius of the inner circle touching the leaflet at the symmetry line ($b_{ri}$) and the bending angle ($b_a$). Again, a bending power $b_p$ is introduced to control the shape of the curve.

#### Sinus and symmetry curve

**Fig. 3:**
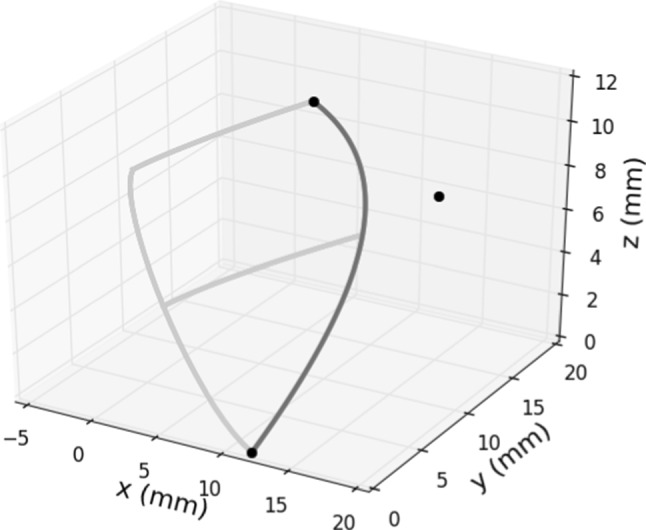
The sinus curve (dark grey) is a Bézier curve defined by the shown control points (black bullets)

The second curve we are adding to the reference model is the *sinus curve* connecting the leaflet, the bending and the annulus planes without defining the sinus structure and the interleaflet triangle. We define this curve from a numerical point of view using a quadratic Bézier curve connecting the three endpoints. The final formula for the sinus curve is the following:

3 $$\begin{aligned} B(t) = (1-t)^2P_0 + 2(1-t)tP_1 +t^2P_2, \end{aligned}$$

where $t \in [0,1]$ and $P_0$, $P_1$ and $P_2$ are the control points (see Fig. 3). $P_0$ and $P_2$ are the start and end point of the curve, respectively, both of them defined using the parameters we introduced before. On the other hand, $P_1$ requires additional information to be defined: we want the sinus curve to intersect the bending curve at the outer circle. With this in mind, we assume that the following equality is valid for $t = s_{sin}$, where $s_{sin} = \frac{b_h}{l_h}$:

$$\begin{aligned} B(s_{sin}) = \begin{pmatrix} b_{ri} \cdot \cos b_a\\ b_{ri} \cdot \sin b_a\\ b_h \end{pmatrix} \end{aligned}$$

Using this equality and plugging $P_0$ and $P_2$ into Eq. (3), we get the following expression for $P_1$:

$$\begin{aligned} P_1 = \frac{B(s_{sin}) - (1-s_{sin})^2P_0 - s_{sin}^2P_2}{2(1-s_{sin})s_{sin}}. \end{aligned}$$

**Fig. 4:**
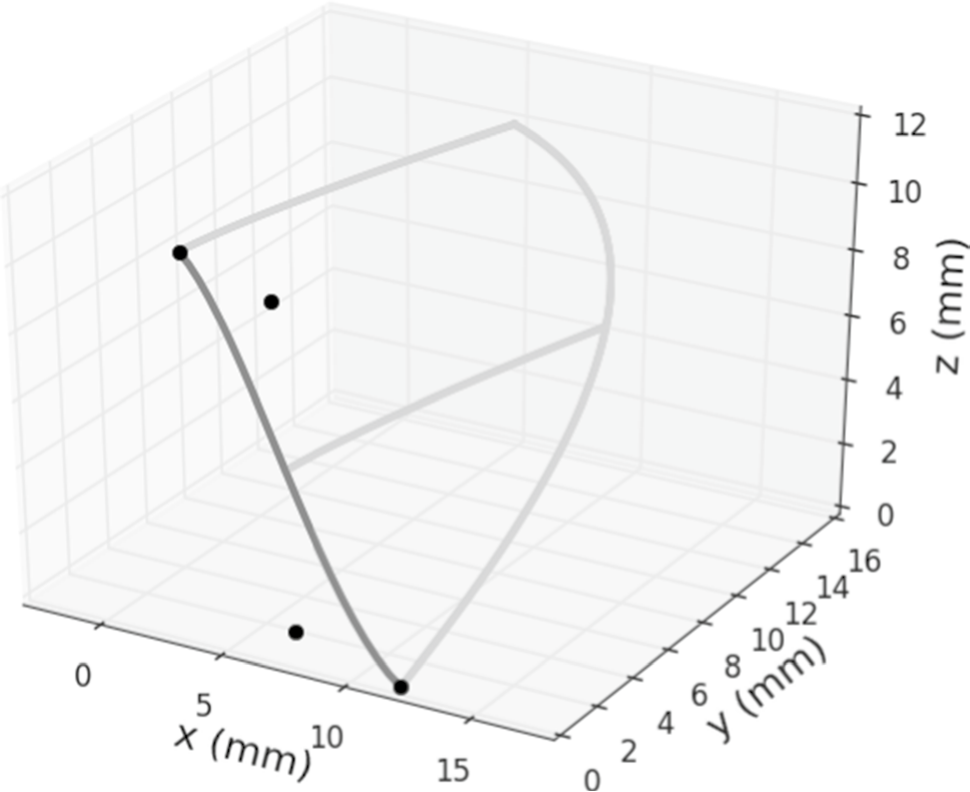
The symmetry curve (dark grey) is a cubic Bézier curve defined by the shown control points (black bullets)

To conclude the description of the leaflet, the *symmetry curve* needs to be introduced. Again, we proceed differently from Haj-Ali et al. (2012), defining the curve as a cubic Bézier curve:

4 $$\begin{aligned} \begin{aligned} C(t) = (1-t)^3 Q_0&+ 3t(1-t)^2Q_1 +\\&+ 3t^2(1-t) Q_2 + t^3Q_3, \end{aligned} \end{aligned}$$

where $t \in [0,1]$ and $Q_0$, $Q_1$, $Q_2$ and $Q_3$ are the control points, also shown in Fig. 4. $Q_0$ and $Q_3$ are the start and end points of the curve and can be defined using the physical parameters as we did for $P_0$ and $P_2$. We set $Q_1$ to capture the tangent direction of the symmetry curve at $Q_0$, so:

$$\begin{aligned} Q_1 = \begin{pmatrix} \frac{r_r + b_{ri}}{2} + xQ^1_{sym} \cdot (r_r - b_{ri})\\ 0 \\ \frac{b_h}{2} + zQ^1_{sym} \cdot b_h \end{pmatrix}, \end{aligned}$$

where $xQ^1_{sym}$ and $zQ^1_{sym}$ are assumed to be equal to $-0.2$. Then $Q_2$ is retrieved analogously to $P_1$, evaluating Eq. (4) at $t=s_{sym}=\frac{b_h}{l_h}$ and assuming:

$$\begin{aligned} C(s_{sym}) = \begin{pmatrix} b_{ri}\\ 0\\ b_h \end{pmatrix}. \end{aligned}$$

### Interpolation of the curves and surface generation

**Fig. 5:**
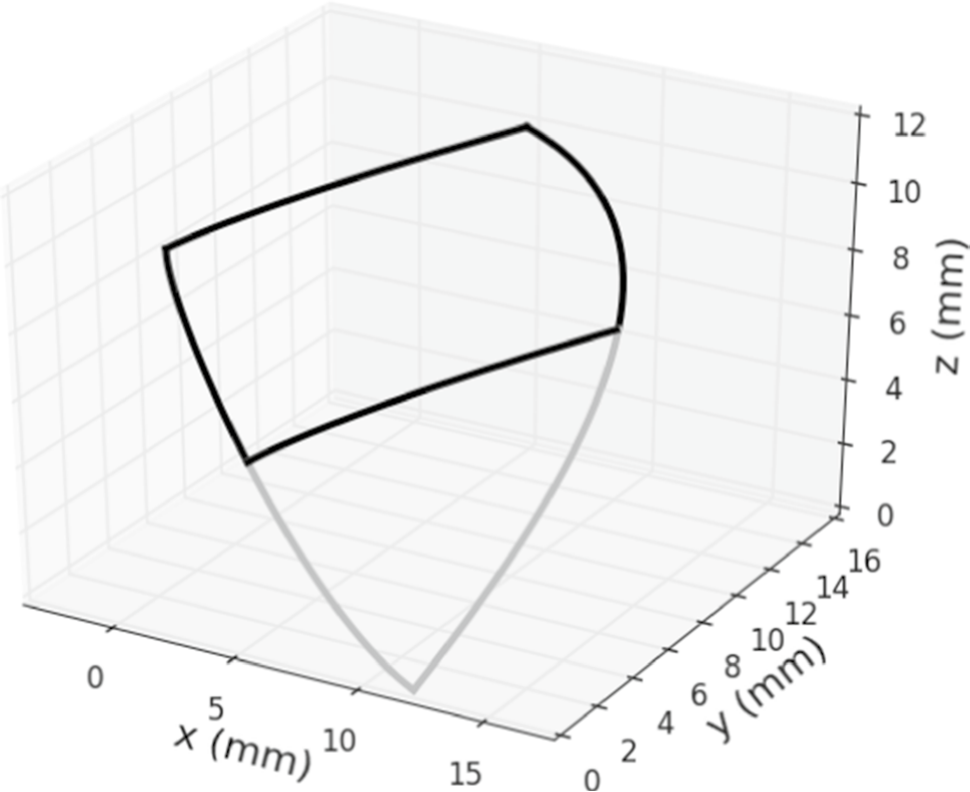
Curves enclosing the top (black) and the bottom (grey) part of the half leaflet

In order to generate the 3D surface of the half leaflet, we divide it into two parts connected smoothly at the bending curve (see Fig. 5). The two analytical curves introduced in the previous section are both approximated with a cubic Bézier curve: to avoid redundancy, we only explain how to choose the control points for the approximation of the leaflet curve, the procedure for the bending one is analogous. We need four control points $L_i$, $i=0,\ldots ,3$, to define a cubic Bézier curve laying on the plane $z=l_h$. While $L_0$ and $L_3$ are simply given by the end points of the analytical curve, $L_1$ and $L_2$ have to be placed in such a way we can control the patient specific shape of the leaflet curve. One way to place them is to use the estimated derivatives of the leaflet curve at $L_0$ and $L_3$. We propose the following expressions:

$$\begin{aligned} L_1&= \begin{pmatrix} L_0[0] + t_0 \frac{f(L_0[1]+\epsilon ) -L_0[0]}{\epsilon } \\ L_0[1] + t_0 \end{pmatrix}\,\text {,} \\ L_2&= \begin{pmatrix} L_3[0] + t_3 \frac{f(L_3[1]-\epsilon ) -L_3[0]}{\epsilon } \\ L_3[1] - t_3 \end{pmatrix}\,\text {,} \end{aligned}$$

assuming for both points $z=l_h$ and *f* defined as in Eq. (1). The weights $t_0$ and $t_3$ are mostly related to the leaflet power and they need to be calibrated in this sense, but in general $t_0,t_3\in (0,1)$. Moreover, notice that the derivative at $L_0$ is equal to zero by construction and the one at $L_3$ changes a lot with the parameters, mostly the power: therefore, more weight is put on the derivative of $L_3$.

Now that all the curves are represented as parametric functions and the symmetry and sinus curves can be easily split into two parts (see Fig. 5) using De Casteljau’s algorithm (see Shirley and Marschner (2009)), the Coons bilinear interpolation (see Shirley and Marschner (2009)) can be done as follows:

5 $$\begin{aligned} \begin{aligned} h(u,v) =&\,\begin{pmatrix} h_1(u,v) \\ h_2(u,v) \\ h_3(u,v) \end{pmatrix}= \\ =&\,(1-v)\cdot \text {bendingCurve}(u) \,+ \\&+ v\cdot \text {leafletCurve}(u) \,+ \\&+ (1-u)\cdot \text {symCurveTop}(1-v) \,+\\&+u\cdot \text {sinusCurveTop}(1-v) \,+\\&- \bigg ((1-u)\cdot (1-v)\cdot Q_{b,sym} \,+\\&+u\cdot v\cdot Q_{l,sin} \,+\\&+ u\cdot (1-v)\cdot Q_{b,sin} \,+\\&+ (1-u)\cdot v\cdot Q_{l,sym} \bigg ), \end{aligned} \end{aligned}$$

where $Q_{ij}$ is the intersection point between curve *i* and *j* and

$$\begin{aligned} Q_{l,sin} =&\begin{pmatrix} l_{ro} \cdot \cos l_a\\ l_{ro} \cdot \sin l_a\\ l_h \end{pmatrix}\text {,} \quad \,\, Q_{l,sym} = \begin{pmatrix} l_{ri}\\ 0\\ l_h \end{pmatrix}\text {,}\\ Q_{b,sin} =&\begin{pmatrix} b_{ro} \cdot \cos b_a\\ b_{ro} \cdot \sin b_a\\ b_h \end{pmatrix}\text {,} \quad Q_{b,sym} = \begin{pmatrix} b_{ri}\\ 0\\ b_h \end{pmatrix}\text {.} \end{aligned}$$

## Application to blood flow simulations

In this section, we include the valve model into the blood flow mathematical model to simulate the flow through subject-specific valves. In order to completely remove the influence of the valve structure on the fluid mesh, we include it in the model using a RIIS approach.

Given a fixed domain $\Omega \subset \mathbb {R}^3$ with its boundary defined as $\Gamma \subset \Omega$, we enrich standard time-dependent Navier–Stokes equations with a penalization term in the momentum conservation equation. This term holds only for the valve surface $\Gamma _{valve} \subset \Omega$ and acts as if we were imposing $\textbf{u} = \textbf{0}$ on the valve leaflets. We also call $\Gamma _{\text {in}} \subset \Gamma$ and $\Gamma _{\text {out}} \subset \Gamma$ the inflow and outflow boundary surface, respectively. The continuous strong formulation of our problem reads as follows: find the velocity $\textbf{u}$ and the pressure *p* such that for each time unit $t \in (0, T_{\text {end}})$ it holds that:

6 $$\begin{aligned} {\left\{ \begin{array}{ll} \rho \frac{\partial \textbf{u}}{\partial t} - \mu \Delta \textbf{u} + \\ \quad \quad + \rho (\textbf{u} \cdot \nabla )\textbf{u} + \nabla p + \gamma \textbf{u}=0 &{} \text {in } \Omega \\ \nabla \cdot \textbf{u} = 0 &{} \text {in } \Omega \\ \textbf{u} = \textbf{u}_{\text {in}}(\textbf{x}, t) &{} \text {on } \Gamma _{\text {in}} \\ \frac{\partial \textbf{u}}{\partial t} = 0 &{} \text {on } \Gamma _{\text {out}} \\ \textbf{u}(\textbf{x}, 0) = \textbf{0} &{} \text {in } \Omega \end{array}\right. } \end{aligned}$$

where $\rho$ is the blood density, $\mu$ is the blood dynamic viscosity, $\textbf{u}_{\text {in}}(\textbf{x}, t)$ is a time dependent parabolic profile and $\gamma$ is a scalar function implicitly defining the valve.

### Numerical approximation and resistive volume extraction

We focus now on the discretization of the resistive model in Eq. (6) and we also detail the pipeline to extract the resistive volume to create the function $\gamma$. The weak formulation of our problem discretized with inf-sup stable elements, already including the Temam stabilization term, reads as follows: find $\textbf{u}_{\text {h}}(t) \in V_{\text {h}}$, $p_{\text {h}} \in Q_{\text {h}}$ such that

$$\begin{aligned} \begin{aligned} \rho \int _{\Omega }\dot{\textbf{u}}_{\text {h}}\cdot&\textbf{v}_{\text {h}}\, d\Omega \,+ \rho \int _{\Omega }(\textbf{u}_{\text {h}}\cdot \nabla )\,\textbf{u}_{\text {h}}\cdot \textbf{v}_{\text {h}}\,d\Omega \,+\\ {}&+ \frac{\rho }{2}\int _{\Omega }(\nabla \cdot \textbf{u}_{\text {h}})\,\textbf{u}_{\text {h}}\cdot \textbf{v}_{\text {h}}\,d\Omega \,+ \\&+ \mu \int _{\Omega }\nabla \textbf{u}_{\text {h}}\cdot \nabla \textbf{v}_{\text {h}}\,d\Omega \,+\\&- \int _{\Omega }p_{\text {h}}\nabla \cdot \textbf{v}_{\text {h}}\,d\Omega + \int _{\Omega }\gamma \textbf{u}_{\text {h}}\cdot \textbf{v}_{\text {h}} \,+ \\&+ \int _{\Omega }q_{\text {h}}\nabla \cdot \textbf{u}_{\text {h}}\,d\Omega = \textbf{0} \end{aligned} \end{aligned}$$

with $\textbf{u}_{\text {h}}=\textbf{u}_{\text {in}}(t)$ on $\Gamma _{in}$ and $\textbf{v}_{\text {h}}=\textbf{0}$ on $\partial \Omega$, $\forall (\textbf{v}_{\text {h}},q_{\text {h}})\in V_{\text {h}}\times Q_{\text {h}}$, where $V_{\text {h}}$ and $Q_{\text {h}}$ are respectively defined as follows:

$$\begin{aligned} V_{\text {h}}\subset V&= [H^1_{\Gamma }(\Omega )]^3=\\&=\{\textbf{v} \in [H^1(\Omega )]^3 : \textbf{v} = 0 \text { on } \Gamma \}\,\text {,}\\ Q_{\text {h}}\subset Q&= L^2(\Omega )\,\text {.} \end{aligned}$$

Such formulation is further stabilized with a suitable backflow stabilization term to avoid nonphysical numerical instabilities at the outlet for high Reynolds numbers (see also Bertoglio and Caiazzo (2016)). Time discretization has been performed using backward Euler method.

Finally, we need to define the function $\gamma$ in the penalization term. We decided to create a scalar function defined on the nodes of the fluid mesh to implicitly define the valve structure after defining all the leaflets of the aortic valve. The parametric model we introduced in Sect. 2 can be used to create a resistive volume of the valve itself to be included in blood flow simulations. Starting from the description of half a leaflet, the construction of the tricuspid valve is straightforward. Consider again the highlighted configuration in Fig. 2e. Leaflets are composed by two identical halves, so a simple transformation by symmetry with respect to the x-axis allows us to recover the complete structure of a single cusp. Our algorithm recovers each leaflet in this position and then applies a rotation by an angle $\theta$ extracted from the CT images to relocate the cusp in the correct position. In this way we obtain a complete geometric subject-specific model of the valve that can be included in our simulations.

After recovering the complete valve, the last step of this pipeline is the actual creation of a resistive volume consisting in a piecewise linear scalar function $\gamma$ defined on the nodes of our mesh. Consider the expression in  Eq. (5). Each leaflet is defined by 2 parametrized functions, therefore the valve structure is indeed defined by 6 functions, namely:

$$\begin{aligned} h^i(u,v) = \begin{pmatrix} h_1^i(u,v) \\ h_2^i(u,v) \\ h_3^i(u,v) \end{pmatrix}\text {,} \;i=1,\dots ,6\,\text {.} \end{aligned}$$

For each node $N = (x_1^N, x_2^N, x_3^N) \in \Omega$, we compute the minimum 3D distance *d* to the valve structure by solving the following minimization problem with a Powell minimization method:

$$\begin{aligned} \min _{\begin{array}{c} u \in (0,1) \\ v \in (0,1) \end{array}} \, \min _{i=1,\dots ,6} \,\left( \,\sum _{j=1}^{3} \bigg (h^i_j(u,v)-x^N_j\bigg )^2\,\right) ^{\frac{1}{2}} = d \end{aligned}$$

and we assign the node a sufficiently large constant value $C \ne 0$ (in our case, $C = 10^{8}$) if it is close to the valve surface, hence:

$$\begin{aligned} \gamma (N) = {\left\{ \begin{array}{ll} C &{} \text {if } d\le \varepsilon \,\text {;} \\ 0 &{} \text {otherwise}\,\text {.} \end{array}\right. } \end{aligned}$$

The value of $\varepsilon$ is chosen accordingly with the mesh size to obtain a sufficiently thin structure, in our case we chose it to be 75% of the maximum mesh size, $h_{\text {max}}$. This choice is a good compromise to have leaflets with a reasonable thickness to ensure a correct recovery of the pressure jump through the valve and to avoid undesired holes in the leaflet surfaces, since decreasing the value of $\varepsilon$ increases the possibility that some nodes in a reasonable proximity of the theoretical surface are assigned the value zero. In case of further refinement of the mesh, we can fix such value so that the thickness of the valve will be not less than the physiological thickness, $h_{\text {phys}}$. In this way, we get the following definition of $\varepsilon$:

$$\begin{aligned} \varepsilon = \max \bigg (\frac{h_{\text {phys}}}{2}, \, 0.75\cdot h_{\text {max}} \bigg )\,\text {.} \end{aligned}$$

## Numerical examples and discussion

We performed blood flow simulations on a set of 10 patients with a monolithic velocity-pressure coupling, backward Euler time discretization and $\mathbb {P}^1_{\text {bubble}}$/$\mathbb {P}^1$ velocity/pressure finite elements. For each patient, we created a personalized valve structure using our model and we solved the fluid-dynamic problem in a cylindrical mesh with radius $r=~l_{ro}$ and average mesh size $h_1=~0.5\,\text {mm}$ in the valve region and $h_2=~0.75\,\text {mm}$ elsewhere. The inflow boundary condition is a time dependent parabolic profile defined over the approximated systolic phase interval $T=~0.4\,\text {s}$ and we run all the simulations up to the peak, hence $T_{\text {end}}=~0.2\,\text {s}$. The maximum Reynolds number at the inlet is fixed to $Re=~1200$ for all the examples, independently on the severity of the aortic stenosis. We assumed the valve to have a fixed open configuration.

**Fig. 6:**
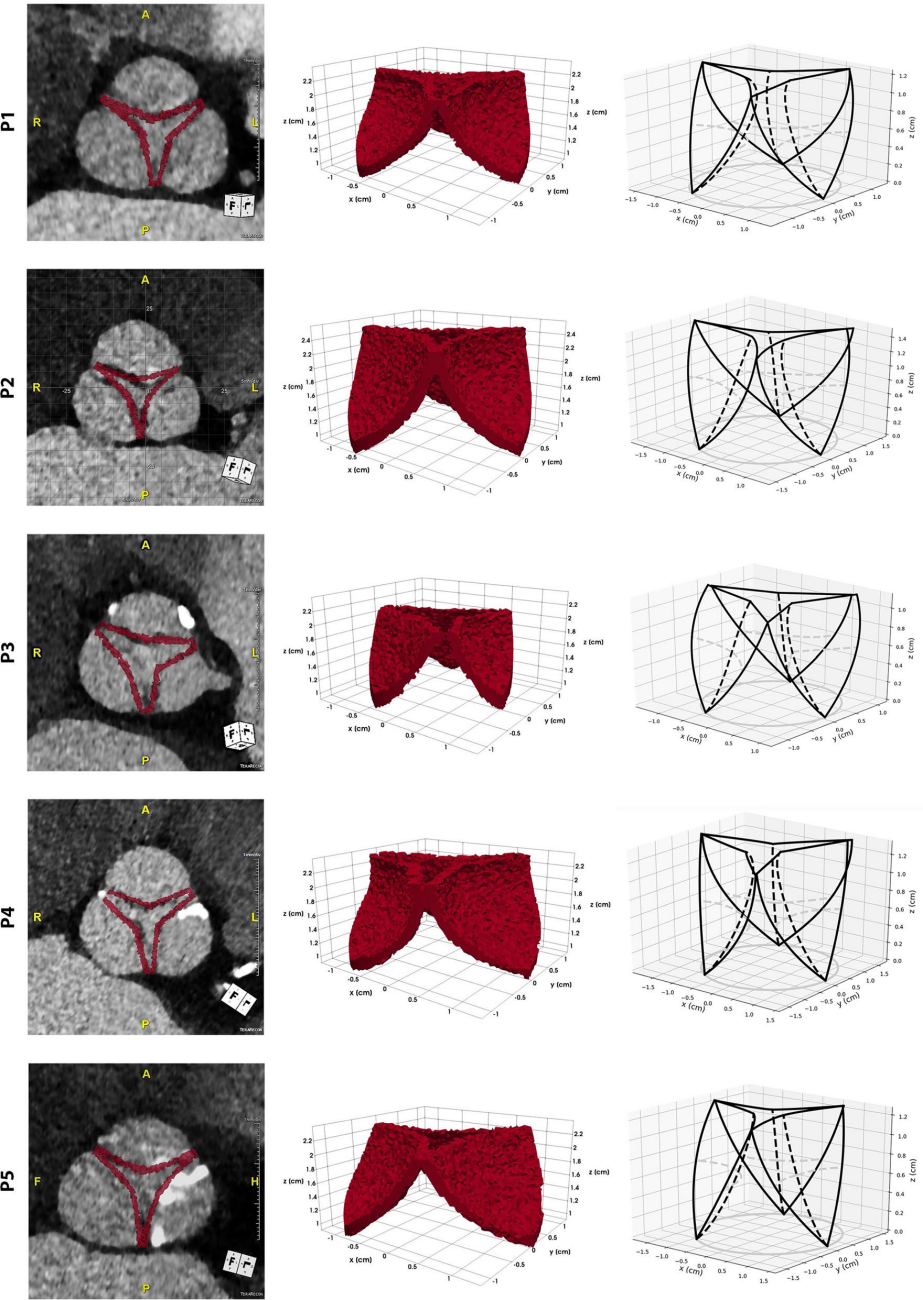
The valve models of the first 5 patients are shown here, one in each row: a qualitative comparison between the obtained geometry and the CT images (left column), the function $\gamma$, suitably thresholded to show only the nonzero values (central column), and the theoretical 3D valve structure (right column)

**Fig. 7:**
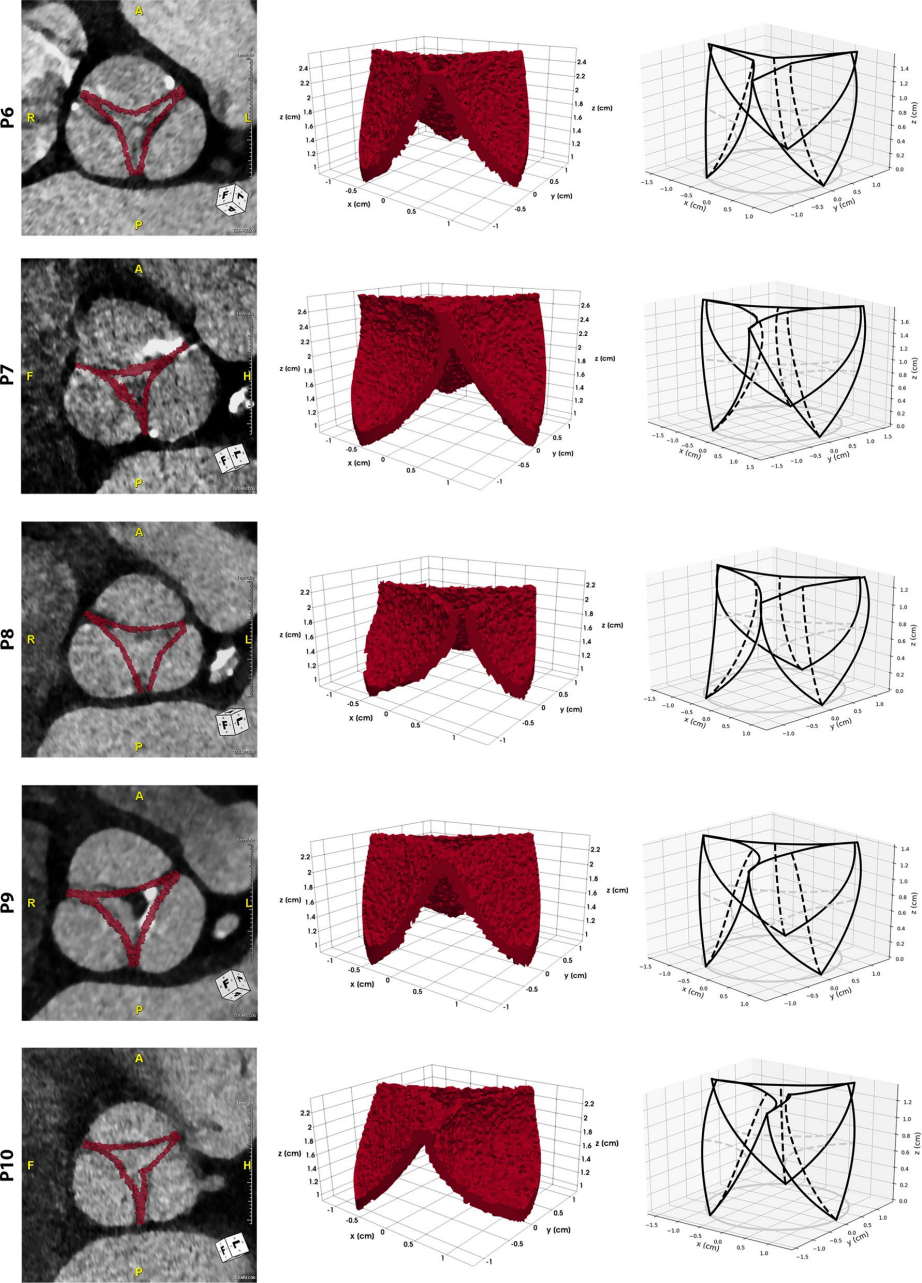
The valve models of the last 5 patients are shown here, one in each row: a qualitative comparison between the obtained geometry and the CT images (left column), the function $\gamma$, suitably thresholded to show only the nonzero values (central column), and the theoretical 3D valve structure (right column)

These experiments allow us to both validate our model by qualitatively comparing it with the images and to observe the effects of a patient-specific pathological valve on the blood flow around the valve region. Figures 6 and 7 show the valve models we obtained for the patients in our data set. Anatomical valves are highly non-symmetric and may vary a lot from patient to patient in shape, size and severity of the stenosis. For each patient we present two different 3D views of the valve structure and a qualitative comparison with the medical images. As described in Sect. 3.1, the pipeline to obtain a valve structure ready to be included into the numerical simulations consists in two separated parts. The first step is to create a personalized model, generating all the 3D parametric surfaces defining the leaflets, hence obtaining the theoretical model that can be observed in the right column of Figs. 6 and 7. Once we generate the model, we use it to compute the function $\gamma$: in the central column of Figs. 6 and 7 we reported the nonzero values of the functions $\gamma$ we obtained from the geometric model. In all the cases, the leaflet is 3/4 elements thick, so approximately 2 mm at most. In this model the leaflet thickness is assumed to be homogeneous but this is only an approximation of the actual leaflet distribution, which is thicker on the free margins and attachments edges and thinner in the belly. We can observe that our model is able to cover a wide variety of valves, with very different leaflets in both size and shape. The parametrization we made allows us to recover also a wide range of stenotic, and for this reason very irregular, orifice areas. A further qualitative validation of this pipeline has been made by overlapping the resulting model to the CT images (Figs. 6 and 7, left column). As expected, the main consequence of our initial assumptions is the over-regularization of the intra-symmetric leaflets structure, and for this reason still different from the anatomical valve in some cases. Despite this fact, the commissures coincide and the variability in terms of shape is well recovered even if we relaxed the inter-symmetry assumption.

Concerning the fluid-dynamic simulations, we can exploit the flexibility of our model to investigate the valve shape induced blood flow patterns. Velocity results are reported in Figs. 9 and 10. For each patient, we show the velocity magnitude contours on a long-axis slice of the domain at peak systole (left) and a 3D representation of the flow through the valve (right). Due to the partially obstructed aortic orifice, a high-velocity jet forms after the valve and the velocity magnitude increases with the severity of the stenosis. As expected, the stenotic valve shape influences the direction of the blood jet and for some patients it also causes an abnormal impinging jet against the cylinder wall. The streamlines view gives also a general idea of how the flow actually develops throughout the domain: the correct implementation of the resistive term forces the blood to move along the valve surface, then contributing to the formation of the previously described jet. In some of the more open cases we can also notice some recirculation vortices (e.g., case P3 and P8). In Fig. 11, we show the pressure results on a slice for the 10 patients at peak systole. The pressure field is characterized by a high gradient across the valve level and it is almost homogeneous elsewhere. Such gradient increases in time in the first half of the systolic phase reaching their maximum at the end time of the simulation and tends to increase as the severity of the stenosis increases.

**Fig. 8:**
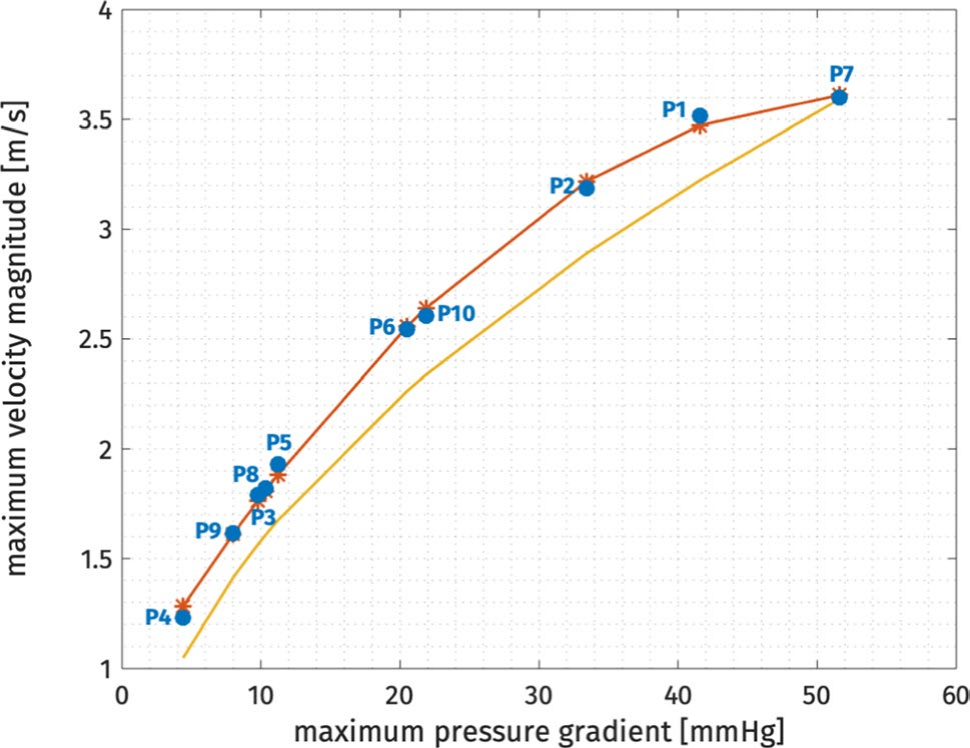
The relation between pressure gradient and maximum velocity is reported in this picture. The red curve represents the second-order polynomial fitting of our data (blue bullets). The yellow curve represents the theoretical relation obtained using the simplified Bernoulli equation

In clinical practice, measuring the pressure gradient across the aortic valve is a challenging task and it may also require invasive procedures. The standard way to estimate it consists in recovering it from the velocity data acquired from Doppler imaging using the simplified Bernoulli equation $\Delta P = 4 \, v_{max}^2$ (see Donati et al. (2017)). Such relation can be investigated using the results of the numerical simulations. We computed the maximum velocity magnitude and the maximum pressure gradient for the patients in the data set and we reported them in Fig. 8. The result of the fitting of our datapoints is a quadratic polynomial (reported in red in the picture) which is always greater than the Bernoulli equation approximation (yellow curve in the picture). This may be an indicator that the simplified Bernoulli equation tends to underestimate the severity of the stenosis, but this claim needs further investigation on a much larger sample to be adequately proved.

**Fig. 9:**
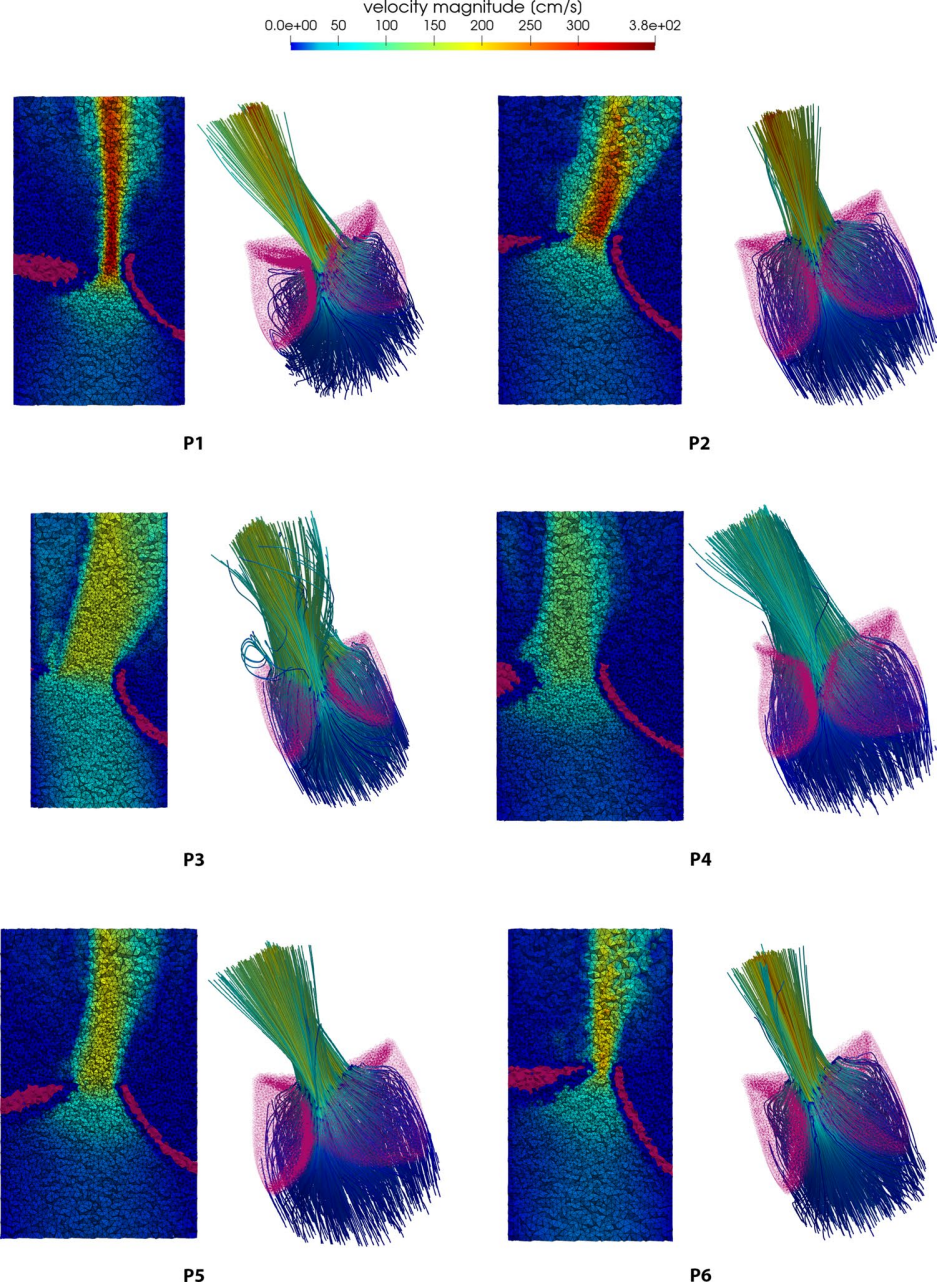
Velocity results of the blood flow simulations through the aortic valve for the first 6 patients at peak systole: for each patient, we show the velocity contours on a slice of the domain (on the left) and a 3D representation of the flow through the valve (on the the right)

**Fig. 10:**
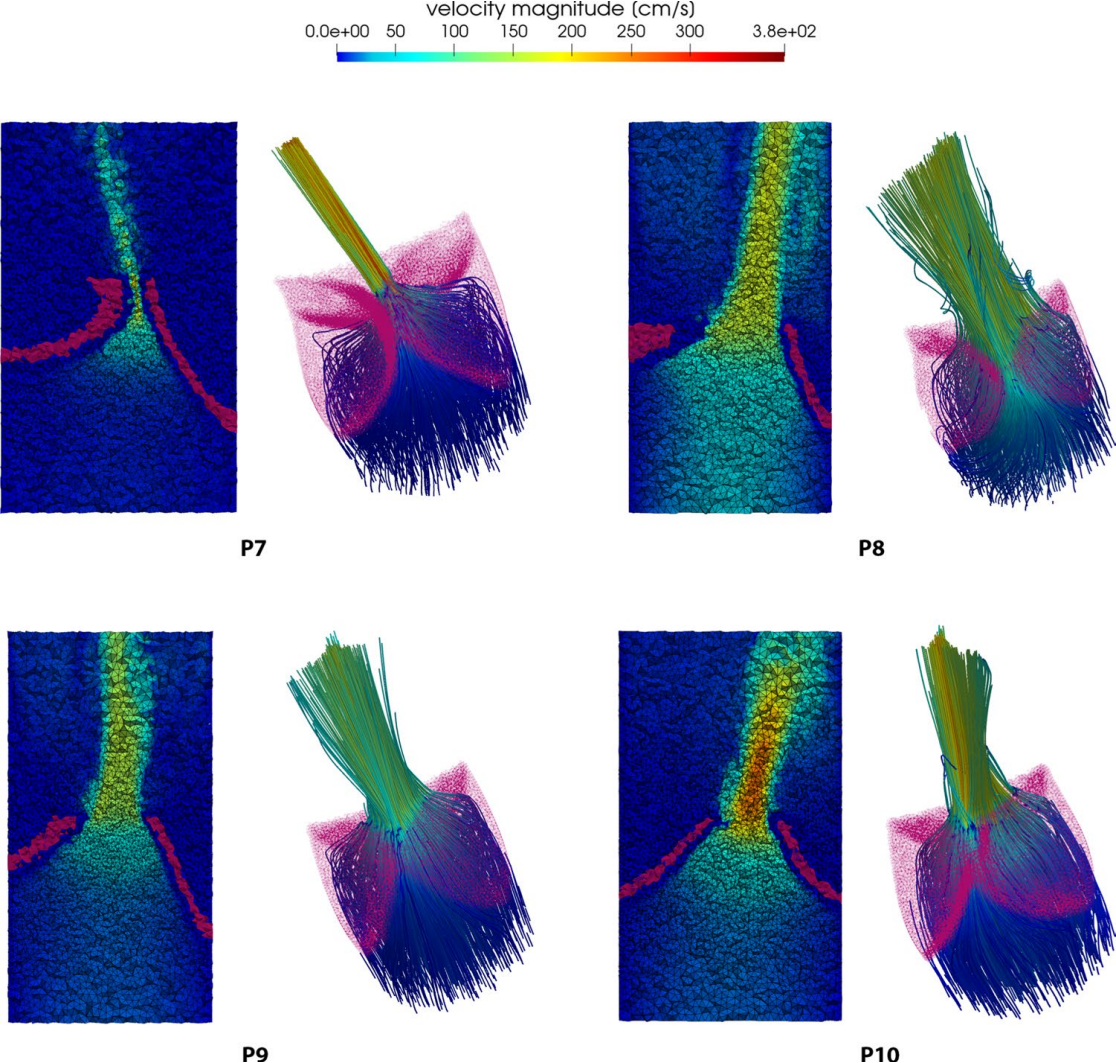
Velocity results of the blood flow simulations through the aortic valve for the last 4 patients at peak systole: for each patient, we show the velocity contours on a slice of the domain (on the left) and a 3D representation of the flow through the valve (on the the right)

**Fig. 11:**
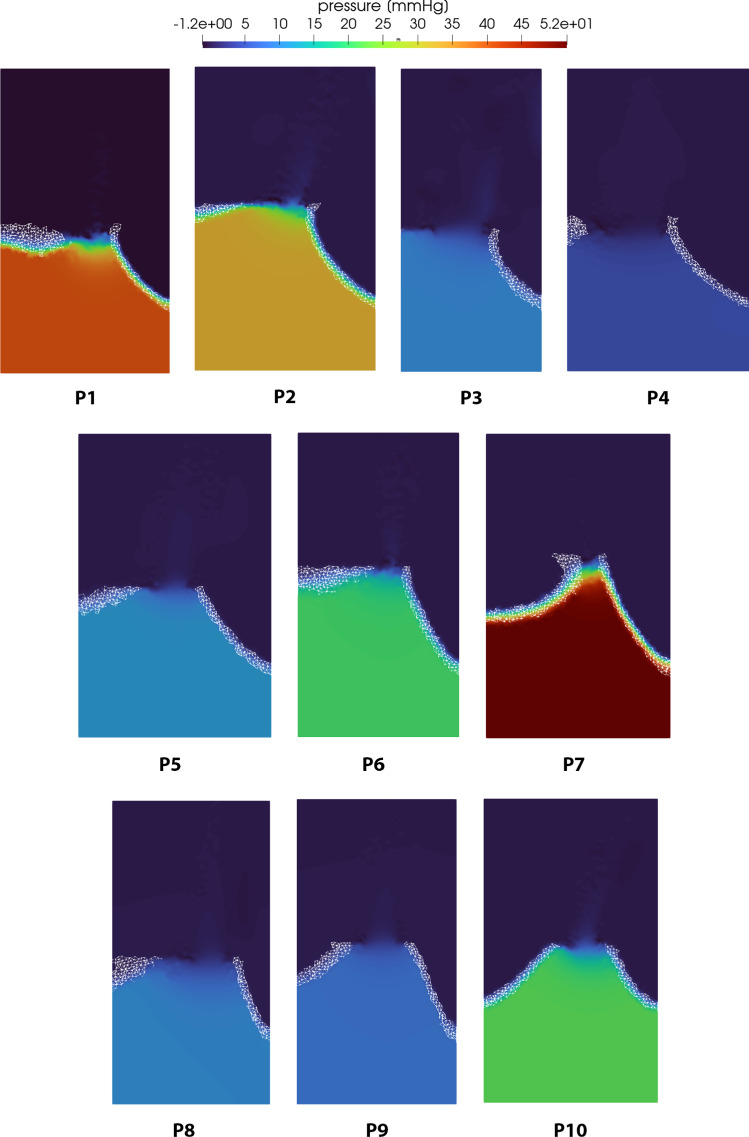
Pressure results of the blood flow simulations through the aortic valve at peak systole for all the 10 patients in the dataset

## Conclusions

This work represents a first approach to the use of personalized parametric valve models in pathological cases to study the fluid-dynamic induced by the valve in the aorta. In this paper we presented an improved parametric model of the aortic valve, suitable for both healthy and stenotic valves, generalizing the model presented in Haj-Ali et al. (2012) and relaxing the inter-symmetry assumption. Moreover we also proposed a short pipeline to include the aortic valve into blood-dynamics simulations using a RIIS approach and we discussed application examples on a sample of 10 patients.

The improved valve structure has been successfully included into fluid-dynamic simulations. However, it still presents some limitations. For some sets of parameters we noticed a poor recovery of the commissures which leaves the leaflets disconnected. This problem may rely in some errors in the parameters extraction but also in the fact that we decided not to recover the sinuses for these first experiments, affecting negatively the reconstruction of that region. Running the simulations in an anatomical domain (including the sinuses and the ascending aorta) could almost completely solve the open commissures issue and also improve the fluid-dynamic results. Moreover, even if leaflets often present irregularities in their shape, the modeling results obtained are overall satisfactory in terms of shaping, sizing and approximating the stenosis and give a coherent fluid dynamic pattern for both velocity and pressure fields. Finally, the quadratic relation between the transvalvular pressure gradient and the peak velocity in our dataset confirms the reliability of the implementation and opens up to possible further investigation and related clinical studies.

## Figures and Tables

**Fig. 12:**
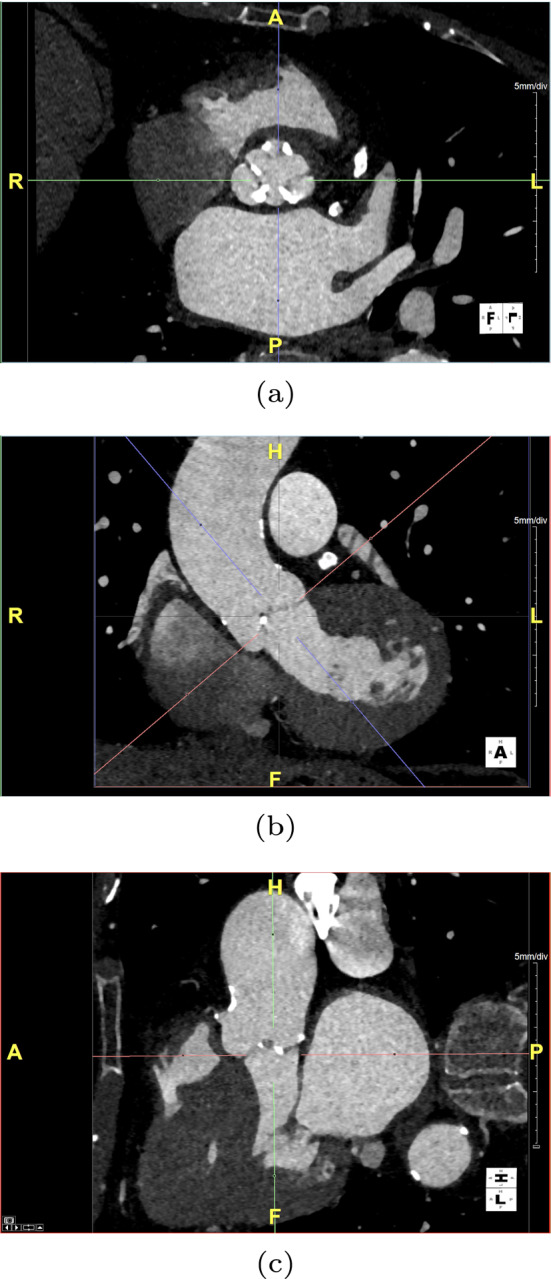
Aligned visualization panels in TeraRecon: xy-plane (a), yz-plane (b) and xz-plane (c)

**Fig. 13:**
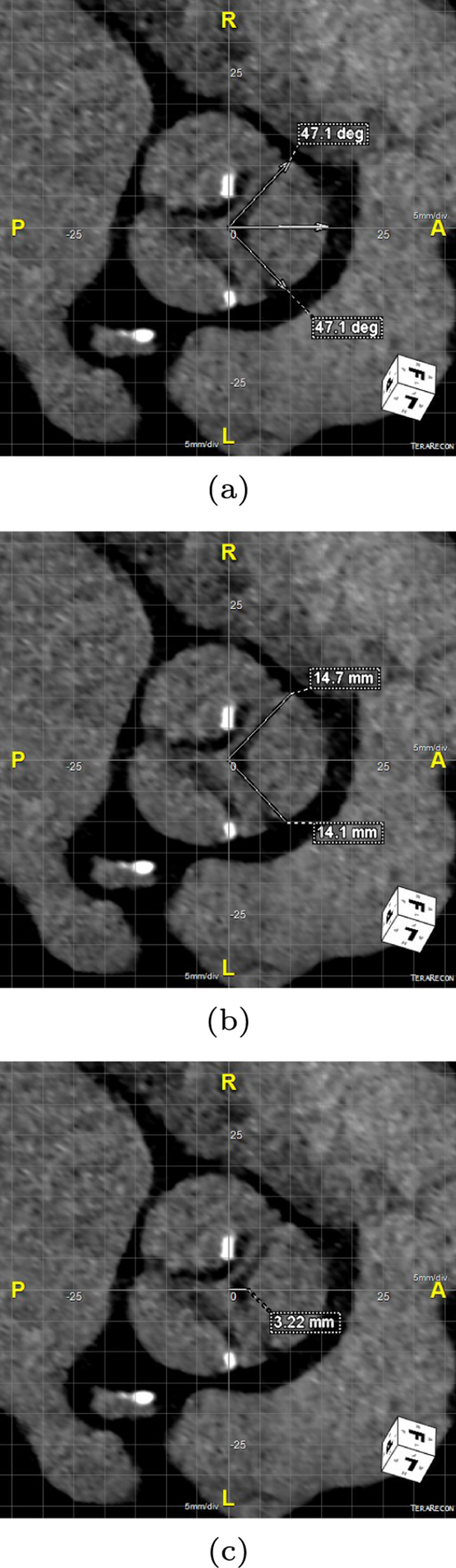
Example: measurements at the leaflet plane to get the leaflet angle (a), the outer radius (b) and the inner radius (c)

**Fig. 14:**
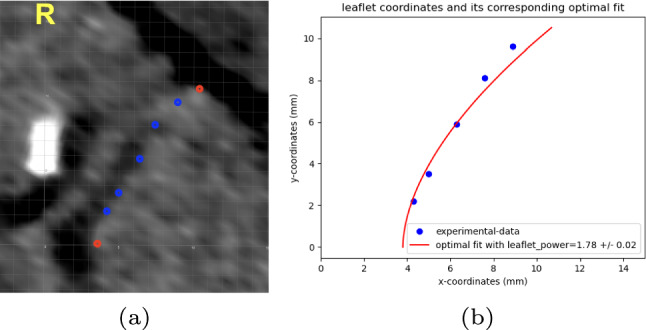
Example: power determination. On the left the points selection is shown (blue), together with the boundary values that must be intersected by the fit. On the right the result of the fitting (red curve) and the extracted points (blue) are reported

## Appendix A: Patients data set and parameters extraction

The dataset used in this study has been selected among subjects diagnosed with aortic stenosis. As candidates for a Transcatheter Aortic Valve Implantation (TAVI), ECG-gated pre-surgery CT scans are available for all the subjects. We use the biomedical imaging software TeraRecon to analyze the images and extract the shape parameters we need for the model.

After aligning the visualization panels to our reference system (see Fig. 12), we need to fix the axes origin in the center of the lowest part of the aortic root. A good placement of the origin is crucial to accurately extract the parameters. Due to the lack of inter-symmetry in our model, a way to approximate its position consists in drawing the symmetry line of each cusp in the leaflet plane and then using the center of the triangle they form as the center of our reference system.

We then proceed with the extraction of the shape parameters. Since the aortic annulus has a non-regular shape, we use the TeraRecon built-in tool *assisted distance pair* to compute the average diameter of the aortic annulus area and so to measure the $\mathtt{annulus\_radius}$. Sliding the xy-plane along the z-axis, we can measure the $\mathtt{leaflet\_height}$, defined as the distance between the top part of the valve and the annulus plane, from which we get the $\mathtt{bending\_height}$, defined as half the leaflet height. On the bending and leaflet plane, all the leaflet-related shape parameters can be measured. This process is the same for the 3 leaflets in both planes. For both of them, we place an oriented grid on the image in such a way that the x-axis of the grid splits the leaflet into two halves, so that the $\mathtt{leaflet\_angle}/\mathtt{bending\_angle}$ are immediately given (see Fig. 13, left). On the same planes we can also measure the $\mathtt{leaflet\_radius\_out}/\mathtt{bending\_radius\_out}$, defined as the average of the distances between the origin of the grid and each of the commissures (Fig. 13, center), and the $\mathtt{leaflet\_radius\_in}/\mathtt{bending\_radius\_in}$, i.e., the distance between the origin of the grid and the leaflet in the direction of the x-axis (Fig. 13, right).

The last two parameters, the $\mathtt{leaflet\_power}$ and the $\mathtt{bending\_power}$, cannot be extracted directly, since they describe to what extent the leaflet bends. As these shape parameters are the last unknowns in (1) and (2), they can be obtained by finding the optimal fit of a set of points selected on the image using a least squares method. The coordinates of a set of 5 points on the leaflet/bending curve are extracted from the CT-scan. Since we have an error in both the x- and y-coordinate, an orthogonal distance regression is chosen to be the best least squares method to approximate the powers: an orthogonal distance regression minimizes the sum of the square distances perpendicular to the fitted line. Figure 14 shows how these coordinates are extracted from a CT-scan and how the corresponding optimal curve fits the coordinates.

Finally, to correctly relocate each leaflet in the correct position, a $\mathtt{relative\_rotation}$ angle is measured. This angle is nonzero only for 2 of the cusps (right coronary cusp, RCC, and non-coronary cusp, NCC) and it is obtained by measuring the angle between the corresponding commissures of two leaflets. The complete set of parameters we need to reconstruct a non-symmetrical valve consists of 27 descriptive parameters and 2 additional ones (the relative rotation angles for RCC and NCC), and is reported in Table 1.

**Table 1:** Complete set of shape parameters to reconstruct a non-symmetrical aortic valve

Parameter name	Abbreviation	Dimensions
$\mathtt{annulus\_radius}$	$r_{r}$	[length]
$\mathtt{leaflet\_height}$	$l_{h}$	[length]
$\mathtt{bending\_height}$	$b_{h}$	[length]
$\mathtt{LCC\_leaflet\_angle}$	$l_{a}^{L}$	[angle]
$\mathtt{LCC\_leaflet\_radius\_in}$	$l_{ri}^{L}$	[length]
$\mathtt{LCC\_leaflet\_radius\_out}$	$l_{ro}^{L}$	[length]
$\mathtt{LCC\_leaflet\_power}$	$l_{p}^{L}$	–
$\mathtt{LCC\_bending\_angle}$	$b_{a}^{L}$	[angle]
$\mathtt{LCC\_bending\_radius\_in}$	$b_{a}^{L}$	[length]
$\mathtt{LCC\_bending\_radius\_out}$	$b_{a}^{L}$	[length]
$\mathtt{LCC\_bending\_power}$	$b_{a}^{L}$	–
$\mathtt{RCC\_relative\_rotation}$	$rot_{r}^{R}$	[angle]
$\mathtt{RCC\_leaflet\_angle}$	$l_{a}^{R}$	[angle]
$\mathtt{RCC\_leaflet\_radius\_in}$	$l_{ri}^{R}$	[length]
$\mathtt{RCC\_leaflet\_radius\_out}$	$l_{ro}^{R}$	[length]
$\mathtt{RCC\_leaflet\_power}$	$l_{p}^{R}$	–
$\mathtt{RCC\_bending\_angle}$	$b_{a}^{R}$	[angle]
$\mathtt{RCC\_bending\_radius\_in}$	$b_{ri}^{R}$	[length]
$\mathtt{RCC\_bending\_radius\_out}$	$b_{ro}^{R}$	[length]
$\mathtt{RCC\_bending\_power}$	$b_{a}^{R}$	–
$\mathtt{NCC\_relative\_rotation}$	$rot_{r}^{N}$	[angle]
$\mathtt{NCC\_leaflet\_angle}$	$l_{a}^{N}$	[angle]
$\mathtt{NCC\_leaflet\_radius_in}$	$l_{ri}^{N}$	[length]
$\mathtt{NCC\_leaflet\_radius_out}$	$l_{ro}^{N}$	[length]
$\mathtt{NCC\_leaflet\_power}$	$l_{p}^{N}$	–
$\mathtt{NCC\_bending\_angle}$	$b_{a}^{N}$	[angle]
$\mathtt{NCC\_bending\_radius_in}$	$b_{ri}^{N}$	[length]
$\mathtt{NCC\_bending\_radius_out}$	$b_{ro}^{N}$	[length]
$\mathtt{NCC\_bending\_power}$	$b_{p}^{N}$	–
